# Atlas of Syphilis, and the Venereal Diseases

**Published:** 1899-06

**Authors:** 


Atlas of Syphilis, and the Venereal Diseases, including a brief treatise on the Pathology and Treatment. By Prof. Dr. Franz Mracek, of Vienna. Authorized translation from the German. Edited by L. Bolton Bangs, M. D., of New York. With 71 colored plates. Published by W. B. Saunders, Philadelphia, 1898. Price, $3.50, net.

After what has already been said of the excellence of former volumes of this series of hand atlases, it is obvious that this volume being devoted to the subject of all others most susceptible of being taught by colored illustrations, is of the greatest value. The 71

plates are masterpieces of the engraver's art. They offer a ready and satisfactory substitute for clinical observation, not only of the common forms of venereal disease, but of those rarer forms that so often puzzle us to diagnose. The plates, in this volume, as in the others of the series, are accompanied by excellent and interesting clinical notes *of the cases illustrated,* and are followed by a short, concise and practical treatise on the subject. J.

